# Clinical decision-making and secondary findings in systems medicine

**DOI:** 10.1186/s12910-016-0113-5

**Published:** 2016-05-21

**Authors:** T. Fischer, K.B. Brothers, P. Erdmann, M. Langanke

**Affiliations:** University Medicine Greifswald, Institute for Ethics and History of Medicine, Ellernholzstraße 1-2, 17487 Greifswald, Germany; Kosair Charities Pediatric Clinical Research Unit, University of Louisville, School of Medicine, Louisville, KY USA; Faculty of Theology, Greifswald University, Greifswald, Germany

**Keywords:** Systems medicine, Big data, Epistemology, Translation, Secondary findings, Electronic health records, Scoring systems, Clinical decision-making

## Abstract

**Background:**

*Systems medicine* is the name for an assemblage of scientific strategies and practices that include bioinformatics approaches to human biology (especially systems biology); “big data” statistical analysis; and medical informatics tools. Whereas personalized and precision medicine involve similar analytical methods applied to genomic and medical record data, systems medicine draws on these as well as other sources of data. Given this distinction, the clinical translation of systems medicine poses a number of important ethical and epistemological challenges for researchers working to generate systems medicine knowledge and clinicians working to apply it.

**Discussion:**

This article focuses on three key challenges: First, we will discuss the conflicts in decision-making that can arise when healthcare providers committed to principles of experimental medicine or evidence-based medicine encounter individualized recommendations derived from computer algorithms. We will explore in particular whether controlled experiments, such as comparative effectiveness trials, should mediate the translation of systems medicine, or if instead individualized findings generated through “big data” approaches can be applied directly in clinical decision-making. Second, we will examine the case of the Riyadh Intensive Care Program Mortality Prediction Algorithm, pejoratively referred to as the “death computer,” to demonstrate the ethical challenges that can arise when big-data-driven scoring systems are applied in clinical contexts. We argue that the uncritical use of predictive clinical algorithms, including those envisioned for systems medicine, challenge basic understandings of the doctor-patient relationship. Third, we will build on the recent discourse on secondary findings in genomics and imaging to draw attention to the important implications of secondary findings derived from the joint analysis of data from diverse sources, including data recorded by patients in an attempt to realize their “quantified self.”

**Summary:**

This paper examines possible ethical challenges that are likely to be raised as systems medicine to be translated into clinical medicine. These include the epistemological challenges for clinical decision-making, the use of scoring systems optimized by big data techniques and the risk that incidental and secondary findings will significantly increase. While some ethical implications remain still hypothetical we should use the opportunity to prospectively identify challenges to avoid making foreseeable mistakes when systems medicine inevitably arrives in routine care.

## Background

### What is Systems Medicine?

**Fig. 1:**
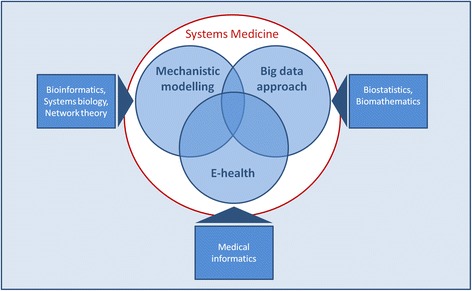
Systems Medicine can be understood as a heterogeneous set of methods and approaches connected by an emphasis on information technologies

A new buzz word has recently entered the discourse on the healthcare of the future: “systems medicine.” This term is used to refer to research approaches intended to improve understanding of biological mechanisms through the use of methods from *omics*-based science, systems biology, bioinformatics and network theory [1–6]. In this respect, systems medicine is closely related to Personalized or Individualized Medicine in that it is an emerging approach that involves the tailoring of prevention, diagnosis, and treatment based on individual patient characteristics [7, 8]. This term is also used, however, to promote a set of related translational practices intended to apply medical informatics tools – such as electronic decision support and patient-collected data – to improve patient care, usually with the secondary aim of making this data accessible for research [9–11]. Both of these meanings share in common a strong focus on the use of information technologies for the purposes of medical science or clinical care. Based on a discourse analysis of the literature on systems medicine [12], then, we can define it as a spectrum of approaches that include (a) bio-mathematical modelling to simulate the mechanistic functioning of biological processes (bottom-up simulation), (b) biostatistical simulation, using hypothesis-free big data-approaches, to model the relationships between the inputs and outputs of biological processes (top-down modeling), and (c) the use of information technologies to synthesize diverse sources of clinical data and automatically generate clinical alerts and guidance (Fig. 1).

The distinction between bottom-up simulation and top-down modelling is a subtle one, but one that is key to our analysis. Bottom-up simulation, utilizing analytical methods from bioinformatics, systems biology, and network theory, is intended to create computer simulations of biological processes at the level of constituent mechanisms, with the hope that such simulations can be used to predict how systems will respond to interventions or perturbations [4, 5]. These methods depend on a causal understanding of the biological mechanisms of interest. For this reason, research groups working within this strain of systems medicine tend to focus on clarifying signaling pathways and molecular pathways that connect, for example, genotypes with phenotypes [13].

Top-down modelling, on the other hand, does not depend on a detailed understanding of biological mechanisms. Instead, top-down models are developed through the statistical analysis of large datasets. This approach is analogous to Amazon’s recommendation engine, which utilizes a huge database of past purchasing behavior to predict which items individuals might want to purchase in the future. This approach, typically referred to as *the big data approach*, relies on statistical associations rather than mechanistic understandings [14]. Amazon’s recommendation engine does not, for example, require an understanding of consumers’ motivations. The recommendations it provides to customers are based instead on the assumption that customers’ future purchasing behavior can be predicted by comparing their past purchasing behavior with that of customers who have purchased similar items. Similarly, top-down models in systems medicine do not begin with signaling pathways or molecular pathways, but rather with large datasets reflecting biological and clinical parameters.

As we will examine in this paper, the relationship between top-down modelling and clinical translation is a matter of some controversy within systems medicine. Some enthusiasts look forward to a day when top-down modelling can be incorporated into clinical information systems to generate useful predictions about individual patients’ health and their response to clinical interventions [15]. Others understand top-down modelling as a hypothesis-generating step that might precede mechanistic research [16, 17] or the identification of promising interventions whose effectiveness would require empirical verification [18].

Within the vision for systems medicine, however, the ultimate goal for both bottom-up simulation and top-down modelling is the development of medical informatics tools. These tools would depend on the collection of large sets of clinical data, including, perhaps, data collected by patients using fitness trackers or other mobile devices. This data would then be used to support clinical decisions, create a long-term electronic “memory” of co-morbidities and other relevant information that could be applied to ongoing treatment, and even be utilized for future research that could be used to optimize these approaches [9, 10].

In short, then, systems medicine is not a single research model or clinical approach. It is a heterogeneous set of practices that have been promoted in recent years under a single name. Perhaps one explanation for this complexity, then, is the nascent state of these sciences. For the most part, applications of systems medicine have not reached clinical contexts with the exception of pilot projects. It is likely that as efforts to apply these ideas to clinical care continue, more clarity and focus will develop.

It would be premature, then, to suggest that we can anticipate or address all of the significant ethical challenges raised by systems medicine. Instead, we will focus here on a few key issues especially relevant to specific components of the field of systems medicine. First, we will examine the compatibility of big data methods with responsible clinical decision-making. This is not an ethical issue proper, but rather an epistemological concern that is likely to give rise to important ethical challenges. Second, we will examine a case study that highlights pitfalls in the application of probabilistic scoring systems to clinical practice. Third, we will examine the challenge of unintended findings that are likely to be raised as the types of medical informatics tools envisioned for systems medicine proliferate.

## Discussion

### Systems medicine and clinical decision-making: the problem of epistemology

Epistemology is the field of philosophy that addresses questions related to knowledge: How do we know what we know? When should we be convinced, and how can we convince others, that new knowledge we have discovered is sound? How can we apply knowledge to real-life problems, such as the application of scientific knowledge to medicine?

Although these questions may seem far removed from research on applying *omics*-based technologies to clinical care, in truth they lie at the heart of ongoing debates within systems medicine. As we have observed, systems medicine is comprised of scientific approaches that are based on mechanistic understanding, which we have called bottom-up simulation, as well as those that are agnostic to mechanisms, which we call top-down modelling. In order to see why this distinction is important, and how it creates barriers for clinical applications of systems medicine, we can examine the same trends within a more mature movement in medicine: personalized medicine.

As we observed earlier, systems medicine overlaps somewhat with the vision for personalized medicine. Both approaches share a focus on identifying statistical associations using large datasets. But while personalized medicine focuses on genotype-phenotype associations, systems medicine additionally draws on other *omics*-based technologies, such as proteomics and metabolomics, as well as broad sources for health data, such as personal mobile devices. One important scientific tool within personalized medicine is the genome-wide association study (GWAS). This type of research involves the identification of genotypes anywhere in the genome that are statistically associated with health-related phenotypes, such as those related to pharmaceuticals dosing. Advocates for personalized medicine disagree about how to apply the knowledge generated by GWAS studies to clinical care [19]. On the one hand, many personalized medicine researchers view genome-wide association study “hits” as the starting point for research into molecular mechanisms; they view GWAS as a hypothesis generating technology [20]. Only once these mechanisms are elucidated could GWAS findings be applied to clinical care. This emphasis on molecular mechanisms can even determine which GWAS findings are considered valid. Some investigators, for example, use understanding of molecular mechanisms to aid in the analysis of GWAS hits, focusing on genetic variants in genes that are thought to be most likely to be causally-related to the phenotype under study.

On the other hand, some personalized medicine researchers do not insist on corroborating evidence derived from research on molecular mechanisms. They advance the idea that genotype-phenotype associations with a large effect size are directly applicable to clinical care. This assumption is most apparent in recent debates on secondary findings, in which some argue that genetic variants can be “actionable” even if the only evidence supporting an association with a phenotype was derived from GWAS studies [21]. This perspective is closely linked with the big data approach, which suggests that top-down models can be effective for addressing real world problems, including recommendations for online retailers as well as health-related interventions.

**Fig. 2:**
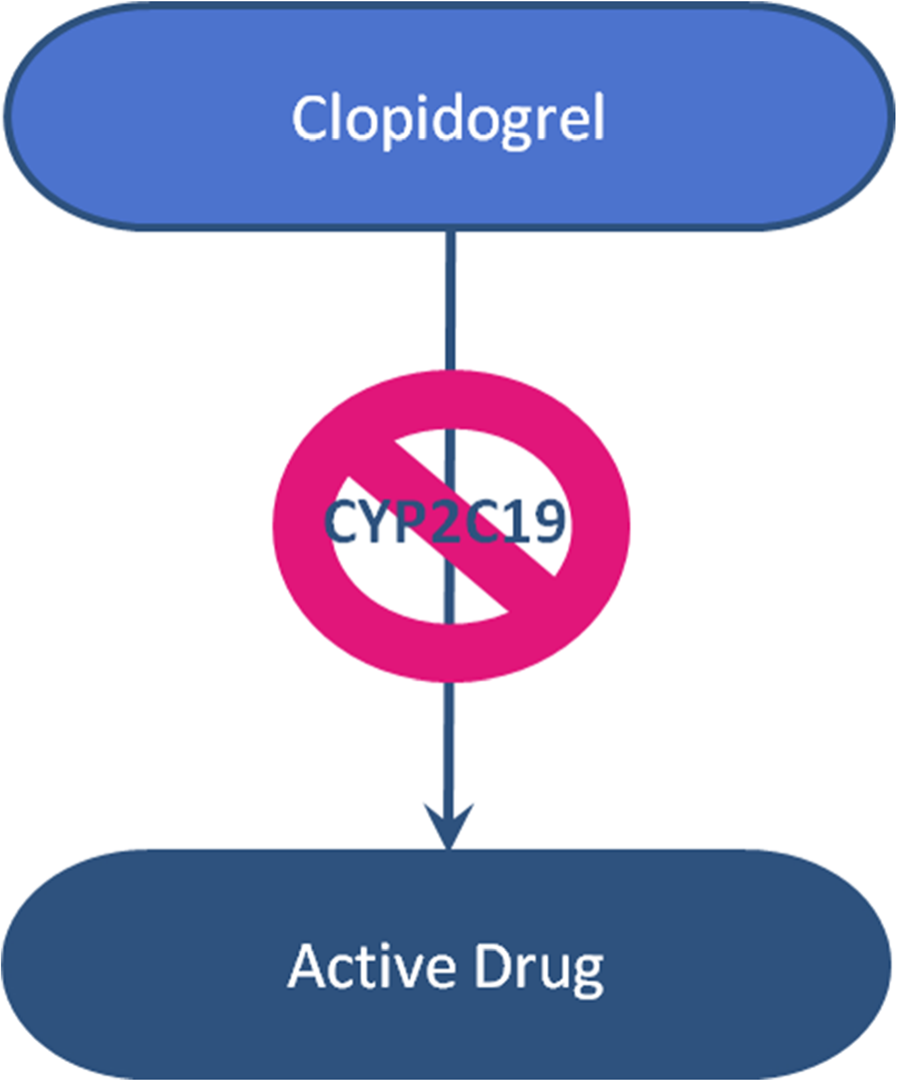
Simplified schema of bioactivation of clopidogrel

However, the application of both bottom-up simulations and top-down models to real world problems can be unexpectedly complex. Within the field of personalized medicine, we can see this in work to apply pharmacogenetic testing to the clinical use of clopidogrel, a drug to prevent blood clots in patients with abnormalities in the cardiovascular system, such as after a coronary artery stent has been placed. The mechanisms by which clopidogrel prevents clots was already well understood: in its active form, it inhibits the adenosine diphosphate P2Y_12_ receptor and therefore inhibits ADP-induced platelet activation. However, clopidogrel is a prodrug meaning that the form contained in a tablet and taken by patients is not active [22]. Before it can effectively inhibit clot formation, it must be bioactived in the body by the cytochrome P450 enzyme produced by the CYP2C19 gene. Some people possess a variant in this gene, however, which reduces the functioning of this enzyme and reduces the bioactivation of clopidogrel (Fig. 2).

For those with such a variant in CYP2C19, we would predict that clopidogrel can provide no benefit. If doctors tested for this variant prior to starting clopidogrel, it was assumed, they could identify patients who would not respond to clopidogrel and instead use a different medication.

**Fig. 3:**
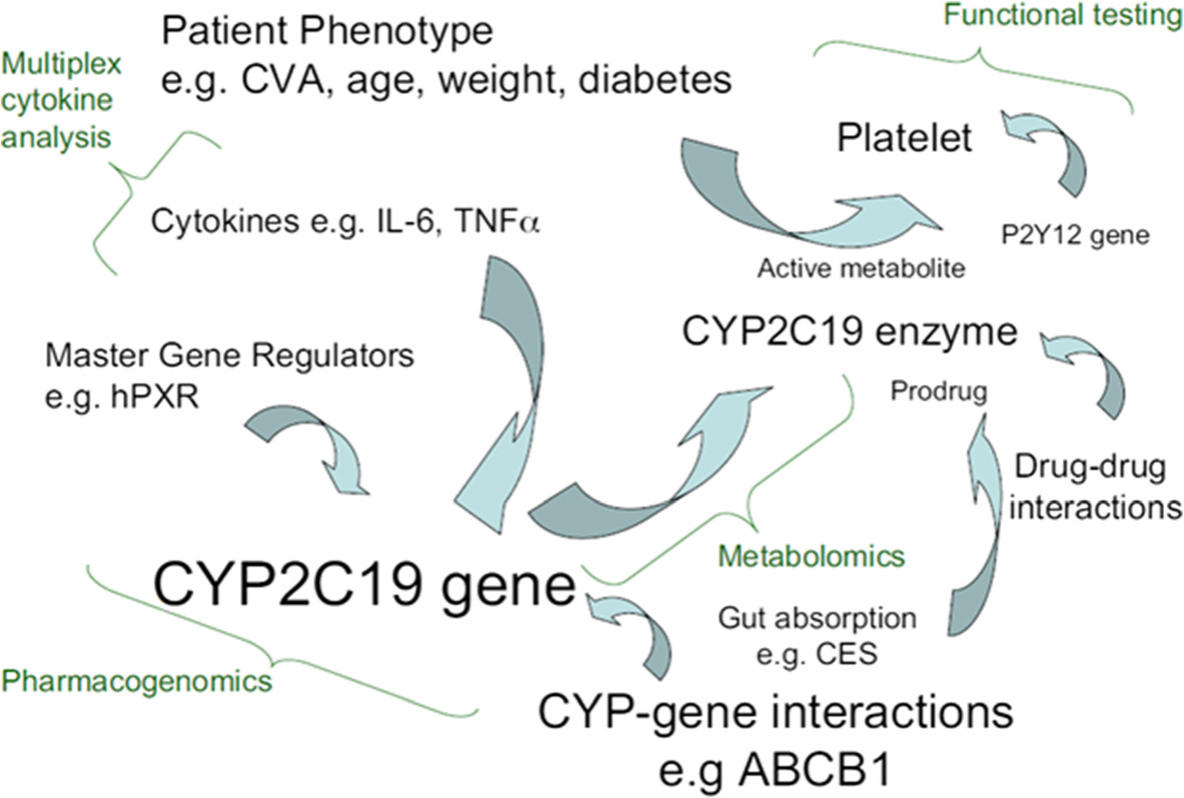
Gladding et al. “Clopidogrel Pharmacogenomics: Next Steps A Clinical Algorithm, Gene–Gene Interactions, and an Elusive Outcomes Trial.” JACC: Cardiovascular Interventions. Oct 2010;3 (10):995-1000. (Reuse of the figure with permission of Elsevier)

Despite this rather well-developed mechanistic model, however, it has proven remarkably difficult to prove that pharmacogenetic testing for CYP2C19 makes a difference in patient outcomes. This is probably because the bioactivation of clopidogrel is not as simple as the model demonstrated in Fig. 2 [23]. A number of other factors influence the degree to which clopiogrel is activated in the body, and its subsequent ability to reduce clot formation (Fig. 3). As evidence from clinical research eventually showed, the model in Fig. 2 oversimplifies the mechanisms that determine whether clopidogrel will work for a particular patient. The biological action of clopidogrel is much more complex than originally thought, and this greatly reduces the utility of testing patients for CYP2C19 variants prior to starting clopidogrel [24].

In this case, scientists working on the pharmacogenetics of clopidogrel made a prediction based on a bottom-up understanding of the mechanisms through which clopidogrel acts in the body. It turned out, however, that this component of the human body, and the way it functions in its complex environment, was more complex than anticipated. It took clinical trials, however, to recognize that our mechanistic understanding was inadequate to guide clinical practice.

This is not to say, however, that bottom-up simulations and top-down models are always unsuitable for application to clinical care. Modern bottom-up simulations simply represents a more mathematical approach to understanding molecular mechanisms, and thus build on a longstanding tradition in medicine that bases clinical care on an understanding of human biology and physiology. This tradition extends back to 19^th^ century physician and laboratory scientist Claude Bernard, and continues today in intensive care units and rare disease clinics around the world [21]. Top-down models, however, have no true precedent in medicine, and thus require closer examination. In the next section, we will examine an example from outside medicine both to further demonstrate how top-down models are different from bottom-up simulations, and to demonstrate why the clinical application of top-down models should be treated with extreme caution.

### The Enigma Machine

**Fig. 4:**
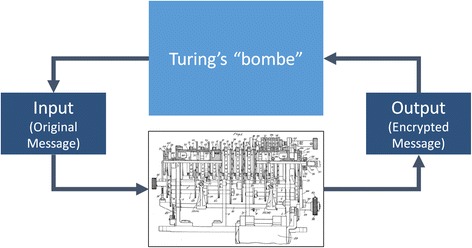
Turing’s solution to decrypting messages encrypted on the Enigma machine

Alan Turing was a British mathematician who worked in the first half of the 20^th^ century. During the second World War, he was enlisted by the British Government to devise a way to interpret messages that had been encrypted using the German Enigma machine. His solution, remarkably ambitious for the time, was to build a simulation of the Enigma machine that could, essentially, run backwards. Although this proved to be an extraordinarily difficult challenge in a world with no digital computers, Turing proved that it was possible by examining the mechanisms the Enigma machine used for encrypting messages. Because British intelligence had managed to recover several Enigma machines, he was able to literally “open the box” and see how it worked. Based on this understanding of how the mechanism worked, he was eventually able to build an early type of computer that simulated the Enigma machine running in reverse [25] (Fig. 4).

In many ways, systems medicine is based on Turing’s work. Later in his career, Turing grew interested in the idea of simulating one computing device using another computing device, even if the underlying mechanisms of the original device were not understood. That this is possible is often referred to as the “Turing-Church Thesis,” and the Turing Test refers to his ideas about how such a simulation could be evaluated.

He argued that a computer could be built to simulate the ability of a human to engage in conversation. He was not suggesting that the entire mind of a human would need to be understood in order to create such a simulation. Instead, he argued that a suitable simulation could be constructed by observing typical human conversations, and would be judged to be effective if it could pass what came to be known as the *Turing Test*. In this test, a human would engage in a dialog with the simulation. If the human could be fooled into thinking he was conversing with another human rather than a simulation, the simulation had passed the test [26].

Turing’s work in both stages of his career serves as an important inspiration for computation-based sciences like systems medicine. In fact, systems medicine includes both a modern equivalent of Turing’s mechanism-based work with the Enigma machine (bottom-up simulation), and the biological equivalent of his later work on simulating the output of the human mind (the Turing Test).

For systems medicine, however, the task of creating a computation-based simulation of the body is not as simple as “opening the box” in the way Turing and other British cryptologists were able to do at Bletchley Park. There are certainly scientists who have this vision for medicine – the vision of understanding the mechanisms of the human body is the focus of all experimental medicine from the time of Claude Bernard to today. But the modern, “big data” approach in systems biology is inspired by the later Turing’s vision. In the top-down approach, the goal is to examine the inputs and the outputs of the human body. The hope is that with enough data, systems biology will be able to generate a computer algorithm that is able to predict how the body will respond to inputs, without actually understanding the mechanisms of the body. For such a model, the internal workings of the body would remain a “black box.” But such an algorithm could, it is hoped, pass a type of clinical Turing test – to imitate the body well enough to guide therapy. Data about an individual patient could be gathered using clinical tests, electronic medical records, and self-monitoring data, and predict a specific output, such as the patient’s response to a variety of possible therapies.

The challenge with this “black box” approach to the human body, however, is that it precludes responsible decisionmaking by patients and healthcare providers.

**Fig. 5:**
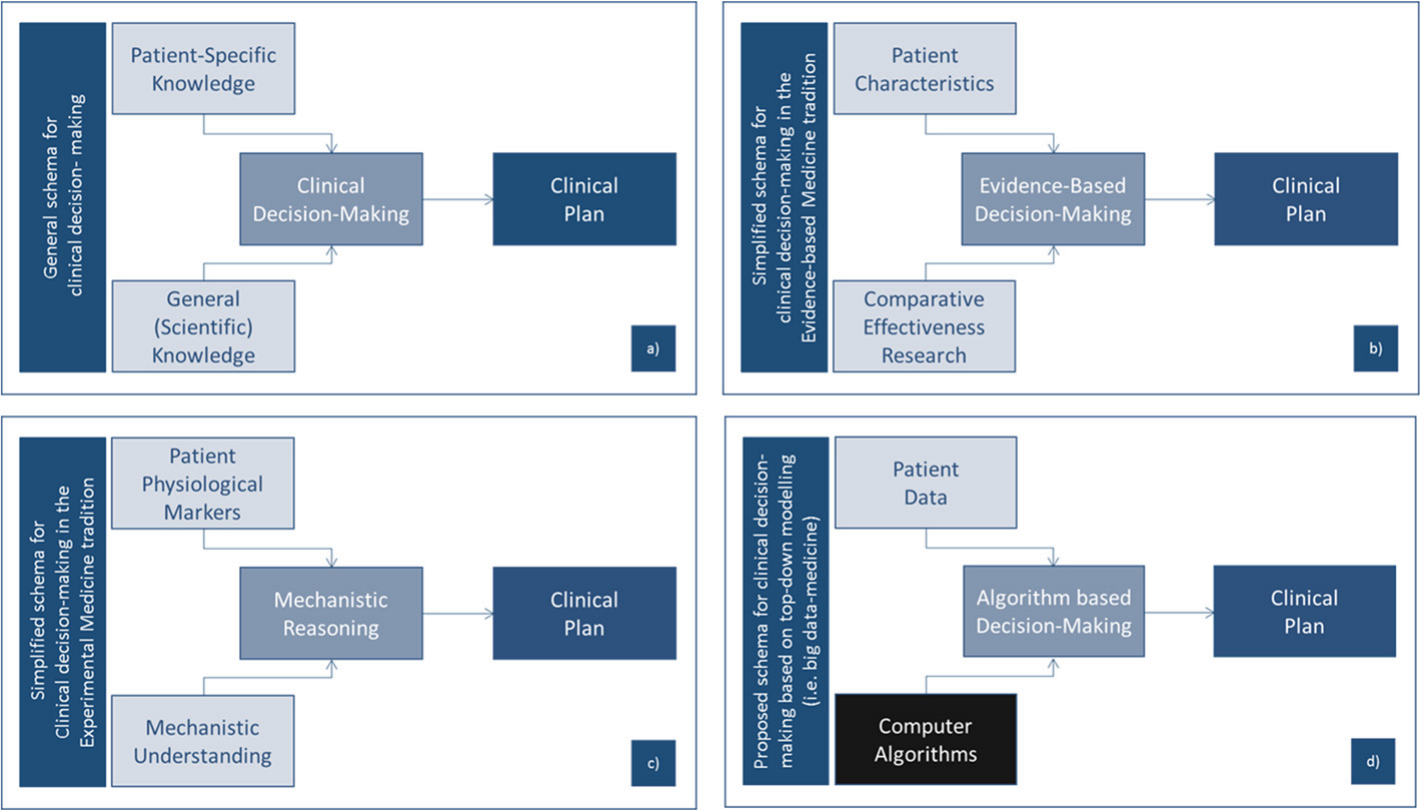
Four approaches to develop a clinical plan

Figure 5a demonstrates how, in general terms, clinical decisions are made. The clinician combines expert knowledge about human biology, social determinants of health, etc. with specific knowledge about the circumstances of a patient in order to develop a clinical plan.

In the tradition of evidence-based medicine (Fig. 5b), clinicians depend primarily on a very particular type of scientific knowledge – empirical findings from clinical research – and particular types of knowledge about the patient. They then utilize a very well-defined process of analysis to apply those clinical research findings to particular patients. Out of this deliberation comes a decision about a clinical plan.

Another prominent approach is the experimental medicine approach (Fig. 5c). In this model, clinicians combine understanding of the mechanisms of the human body with specific observations about the physiological condition of the patient. Using a process of reasoning based on mechanistic cause-and-effect, he or she is then able to develop a clinical plan.

With this in mind, we can begin to see the deep challenge that the top-down dimension of systems medicine poses – and indeed the challenge that many industries face as they work to apply big data analytics to their conventional practices (Fig. 5d). Top-down modelling provides clinicians with a computer algorithm, but that algorithm represents the patient’s body as a black box. It is not based on mechanistic science, and it is not based on clinical research. It simply takes the patient’s data, and generates an output. Neither clinicians nor patients have a way to evaluate its predictions. We are faced with the choice of following its predictions, or ignoring them. But this choice is arbitrary, and based purely on confidence in the black box.

We find this way of applying systems biology to medical practice to be untenable. In order for medical care to be successful, clinicians must be able to be accountable for their clinical decisions, and patients must be given grounds for placing trust in the expertise of their providers. Medical practice must therefore be based on clinical decisions rooted in scientific knowledge, not on the capricious predictions of a black box. Based on this analysis, then, we argue that the top-down dimension of systems biology must be viewed as a basic science – as an approach for generating hypotheses. Whatever potential this approach may hold, it must be mediated by more responsible approaches to medical care, and must therefore not be applied prematurely to clinical care.

In the next section, we will take this critique even further. Taking the Riyadh Intensive Care Program Mortality Prediction Algorithm as an example, we will demonstrate how provider responsibility and patient trust remain significant challenges for systems biology, even when evidence-based medicine has been utilized to validate the effectiveness of predictions based on top-down modelling. In other words, even if this approach works, we are still faced with a complex ethical decision about how and when to use it.

## Ethical aspects of applying scoring systems to clinical care

Within the systems medicine approach, both top-down models and bottom-up simulations are intended for use in clinical care. Specifically, they are designed to combine data about a patient from a variety of sources (medical records, biomarkers, etc.) and apply this data to an algorithm designed to make predictions about how that patient’s illness will progress or how he or she will respond to a particular clinical intervention. Even when these algorithms are based on bottom-up study of biological mechanisms, they are still fundamentally statistical in nature. They are not based on a comprehensive understanding of the patient’s biological status and all mechanisms at play. Instead, they depend on a set of factors that can be measured relatively easily, combined with observations in other patients, to predict which outcomes are most likely.

This approach is fundamentally similar to earlier efforts to develop risk scores based on clinical indicators [16, 17]. Both approaches involve identifying (preferably strong) statistical links between clinical features that can be measured and outcomes of interest, using this data to quantify the likelihood that certain outcomes will occur, and using this score to inform clinical decision-making. Systems medicine simply optimizes this approach by incorporating a larger number of clinical measurements and allowing for more complex scoring algorithms. From an ethical perspective, however, both systems medicine tools and earlier scoring systems carry important implications for clinical practice. In this way, they are not simply pieces of information that are “good to know”. After all, when such scores are generated in clinical settings, an obligation is created for clinicians to respond to them.

### Risk scores in ICUs – The example of APACHE II

As we have seen, an important focus of systems medicine is the generation of statistically-based clinical predictions, predictions that promise to inform clinicians of what is likely to happen, even if they can cast no light on the question of *why* this will happen. One general example of this in a clinical setting is a risk scoring system intended to predict the chances that a patient will survive: “Given all the information we have about patient (P) and correlated with our experience about the outcome of all the patients in similar circumstances for whom we have data in our database, the estimated chance of survival for patient (P) equals X %.” Framed this way, scoring systems can seem quite jarring and impersonal. But in fact, this approach to prognosis is already an important factor of care in intensive care units (ICUs) around the world; and has been for more than three decades. These scoring systems for classifying the prognosis of patients are not based on big data, but rather on more mundane measurements like a patients’ age and routine physiological measurements (temperature, mean arterial pressure, heart rate, respiratory rate, creatinine, hematocrit etc.). While a variety of such scoring systems have been used, one of the best known the APACHE II (Acute Physiology and Chronic Health Evaluation II), which was designed to measure the severity of disease for adult patients admitted to intensive care units [27]. It is designed to be calculated on the day an adult patient is admitted to the ICU, and results in a computer-based score between 0 to 71. Higher scores indicate more severe disease and a higher risk for death. The link between high APACHE II scores, influenced primarily by the presence of multiple physiologic derangements, and risk for dying has been shown repeatedly in independent studies. When applied to the care of a single patient, an APACHE II score essentially generates a prediction about a patient’s risk for morbidity and mortality that is based on the observed rate of outcomes among the group of patients involved in earlier studies who had the same score. APACHE II scores can be used to inform decisions about which treatments should be offered, as well as for creating effectiveness and quality controls [28].

APACHE II is not really intended to generate predictions about an individual patient’s chance for survival. Methods have certainly been developed to support such predictions, but given the wide variety of factors that influence this type of outcome, but are not accounted for in the calculations, such methods are rather imprecise [29–31]. This is just one important reason that it is highly questionable to base decisions about discontinuation of therapy or withholding of interventions based on APACHE II scores and similar scoring systems. Not because these scores are irrelevant to prognosis, but because they generate a prognostic score that is not very precise.

As long as the big data-based scoring systems of the future are regarded as merely updated methods that use more data or more advanced algorithms to make predictions that can be used for similar applications, we would consider it as an improvement with no ethical concerns. However, a brief view back in recent history can provide a preview of the discussions that lay ahead if those in the systems medicine movement come to regard big data-based scoring systems as something more than just a more precise evolution of earlier scoring systems. Specifically, serious ethical questions are likely to arise if such methods are regarded as so precise they should be used to make decisions about withdrawing treatment or withholding life-extending interventions, based solely on the predictive score.

### The Riyadh Intensive Care Unit Program

The Riyadh Intensive Care Unit Program was an effort developed in England in the 1990s that drew its name from the capital of Saudi Arabia, where data from several hundred thousand people had been collected and which formed the statistical basis for the program [32–34]. Unlike other scoring systems like APACHE II, the Riyadh software incorporated not only clinical observations, but also a measure of the intensity of treatment and nursing care that a patient required called the Therapeutic Intervention Scoring System (TISS). The software used these and other measures to generate a so-called “cost-performance profile.” This analysis combined prognostic information (like the APACHE II) and TISS with the intention of evaluating the therapeutic effort (which reflected the cost of treatment) in relation to the chances that an individual patient would survive. By including information outside of that relevant to a patient’s prognosis, the Riyadh program became a cost-benefit analysis. It was intended to suggest when the costs of a given therapy were out of proportion to the patient’s chance of survival; it generated an economic rationale for termination of treatment. Moreover, the Riyadh algorithm was considered to be highly accurate, with an accuracy in its predictions of 99.9 % [32–34].

Following the introduction of the Riyadh algorithm in German ICUs, a debate among physicians, professional societies, ethicists and politicians about the “death computer” ensued. The program was widely critiqued, especially for including economic rationale into the decisions to withdraw treatment [35, 36]. The authors themselves recognized the dangers, and warned: “We consider that the data on outcome predictions with the Riyadh Intensive Care Program have always been presented in a responsible manner. However, we advocate extreme caution in the use of any computer system which predicts death, and advise a careful explanation of its functioning and dangers” [37]. Still, they stuck by the ideas behind their project: “Because of the problems of triage on admission, it is inevitable that a number of patients will be admitted to the ICU with a hopeless prognosis. Treatment of the critically ill with a hopeless prognosis is wasteful of precious medical resources, as well as having a demoralizing effect on the nursing staff and the patients’ relatives” [37].

Should an (expensive) therapy be withheld from patients who are unlikely to survive? Should a computer algorithm be allowed to determine, or even influence, the fate of the critically ill? For this examination, it is not necessary to delve deeper into the ethical debates around the allocation of medical resources or the role cost benefit analyses in medical care. Instead, we simply want to stress that these types of discussions are inevitable once the systems medicine approaches begin to incorporate a greater number of data sources, and lead to the development of algorithms with a higher predictive value. The utilization of non-medical data, up to and including economic data, open the door for abuse or, at the very least, the conflation of medical and non-medical considerations in decision-making. And the perception of greater predictive power inevitably increases the likelihood that such scores will be used as justifications apart from other important considerations, like provider judgments.

Ultimately the Riyadh algorithm failed. The beginning of the end came when further research called the reliability of its predictions into question. The authors of Riyadh found no false predictions of death, but several other investigations identified a survival rate of up to 41 % among those predicted to die [31]. At least one German study has shown that the prognoses of experienced clinicians are at least as good as those generated by the Riyadh algorithm, and another study showed that of 53 patients with “high probability of death,” one third survived. Other important studies have identified a range of problems associated with the application of score-based mortality predictions to individual patients [38].

The Riyadh program never achieved the 99.9 % reliability that was promised. For a time, however, the belief that it could provide highly accurate predictions using all relevant data (including economic data) drove a significant amount of excitement. If, in the coming years, a big data-based scoring system attains the accuracy that was promised for the Riyadh algorithm, we expect these ethical issues will arise again. What role should patient’s wishes play? How do we respect the individuality of the dying life? What weight should be given to considerations of quality of life? What chance for survival should be accepted as for continuing treatment (after all, even if the chance of survival is at 2 %, this still is that 2 out of 100 patients will survive)? What effect should scoring systems have on the doctor-patient relationship, and vice versa? What justifications could be given for rejecting the “objective” prediction generated by an algorithm? What if the score indicates a good chance for survival but the patient does not want to continue treatment?

Even if we were to have access to flawless prognostic algorithms, we would still be left with these larger ethical questions about what to do with this prognostic information. In order to translate prognosis into recommendation, we need to know about the patients’ values [39]. Even in the evidence-based medicine tradition, with its emphasis on basing decisions on empirical data, there is still an emphasis on taking patient preferences and perspectives into account when making clinical decisions [40]. In what ways should systems medicine understand the balance between big data-informed predictions and individual patients’ values, lifestyles, and declarations of will?

At a more fundamental level, we even need to ask whether there is an appropriate role for big data predictions in medical care. Should serious decisions like those related to a change in therapeutic approach or the discontinuation of a therapy be linked with purely statistical interpretations of probability and risks? Or perhaps it is better to assume that the bigger the data and the better the score, the stronger would be the argument for not using it as a sole predictor? Not because of the data for which it can account, but because of the data it cannot. In the final analysis, we are likely to conclude that algorithm-based prediction is never the end, but only the beginning of a decision-making-process. This was even acknowledged by René Chang, one of the key developers of the Riyadh algorithm, defending his software: “If the computer makes a prediction of death, check that there have been no errors in data collection or entry; then determine if there is any treatment which has not yet been tried that might make a substantial difference to the patient’s outcome. If, in the clinicians’ judgment, there is such a treatment, then that treatment should be tried and the patient observed for a significant improvement. If there is no improvement or if in the first instance there were no further treatment regimen available, discussions concerning the withdrawal of treatment should commence with relatives and staff before a final decision is made” [41].

## The ethical dimension of incidental or secondary findings in systems medicine

**Fig. 6:**
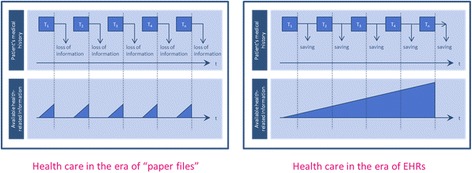
EHRs as electronic “memories”

As shown above, many of the ideas included with the “systems medicine” umbrella depend on the development of medical informatics tools. Among these, of course, are more sophisticated electronic health records (EHRs) that would make it possible for analyses to be performed using stored patient data, including, for example, her co-morbid health conditions. At present, the records of an individual patient’s health and medical care are not routinely stored in such a way that they are simultaneously available for such an analysis. In the future envisioned for systems medicine, however, it would become routine for long-term patient data to be collected and made accessible across computer systems. In this way, every new treatment and new health encounter would add new information to existing patient data. This will eventually lead not only to long term storage of correct (and also incorrect!) diagnoses, but also potentially to an increase in the number of potentially relevant findings about patient’s health that can be generated. In this way, the advances in eHealth envisioned for systems medicine will, almost by design, lead to a continuous and cumulative growth in the number of incidental or secondary findings that can be generated (cf. Fig. 6).

Based on current evidence, two types of medical studies seem especially prone to creating secondary findings in a future medical practice driven by analyses of EHR data: whole body imaging techniques and genomic sequencing [42–47]. Extensive evidence from the research context makes it clear that both of these technologies generate a huge amount of information that is of potential diagnostic, therapeutic or prognostic relevance to patients, or which indicates a need for further examination to determine its relevance [42, 46, 47].

Presently, neither whole body imaging nor genomic sequencing is widely used in clinical care. However, both are already available in research and direct-to-consumer contexts. Someday soon, however, these examinations are likely to be incorporated into clinical care and deposited in patient EHRs, assuming that either (a) data generated in the research or direct-to-consumer context will find its way into the clinical context or (b) these technologies will enter use in clinical settings to inform big data analyses. If this occurs, it is inevitable that this type of data will be applied to analyses not driven by active clinical questions – and thus generate findings that are incidental or secondary to the original reasons the tests were performed. Although these findings might be beneficial to patients, they can also create harms. In one study, for example, we studied participants taking part in a population based-study in Germany that involved whole body MRIs. When this research examination generated suspicious findings, some participants experienced significant harm and distress [42, 43, 46].

**Fig. 7:**
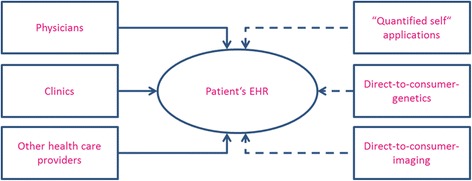
Potential sources for secondary findings

If we consider that even more sources of data will be incorporated into electronic health records – from mobile apps geared toward the “quantified self” to health examinations like psychological analyses and fitness evaluations – we can see that the collection of persistent personal patient information available for analysis is likely to continue to grow. This will lead, however, to significant increase in the frequency and numbers of secondary findings that can be generated (cf. Fig. 7).

If systems medicine proceeds as envisioned, it will not just be the amount of stored patient data that will increase. The vision for systems medicine also includes the analysis of this data for research purposes, leading to an increase in the number of patient characteristics and biomarkers considered to have clinical relevance. Going further, a major goal of big data research is to identify associations among these factors that, in combination, can be used to make clinical predictions, creating a whole additional level of associations and correlations through which secondary findings can be generated.

If these changes take place as predicted, it will become crucial to address a number of important normative questions, including:

1. Who is to decide which findings need to be communicated to providers and patients?
2. By what methods, in a world of big data and patient empowerment, should secondary findings be communicated?
3. How can secondary findings be prioritized responsibly within the treatment context so as to help patients and providers handle a large volume of such findings?

The discourse within human genetics over secondary findings gives us some idea about how these questions will be answered [44, 45]. It is already well-recognized, for example, that it can be difficult for patients and providers to anticipate such findings and prospectively develop a plan for addressing them. When data on the scale of whole genome sequence is generated, such a discussion would require the patient to be informed about the genetic variants that could potentially be identified beyond those relevant to the clinical question motivating the testing, but also understand the various implications for this information and the wide variability in the uncertainty accompanying such findings. Even in Germany, where legal standards are established for the design of such a genetic consultation process, the approaches used for addressing the challenge of secondary findings remains highly inconsistent overall.

The debate over incidental and secondary findings is certainly not new [48]. In human genetics, for example, debates over best practices for informed consent, risk communication, and assessment of clinical validity, among others, predate the current discourse around Personalized or Individualized Medicine [49–51]. In fact, similar debates took place with the field of public health genetics in the 1990s [52]. Nevertheless, systems medicine could provide the context for a range of technological developments that will increase the urgency for identifying effective methods for handling secondary findings and solving some of the difficult questions faced by bioethicists during the last decade and beyond.

## Conclusion

In this paper we examine three possible ethical challenges that are likely to be raised as systems medicine comes to be translated into clinical medicine. These include the epistemological challenges for clinical decision-making created by black box algorithms, the use of scoring systems optimized by big data techniques capable of integrating non-medical data into routine healthcare and healthcare systems, and the risk that incidental and secondary findings will significantly increase as big data-driven EHRs are implemented in clinical care. At this point in time, all three of these concerns are somewhat conjectural. We might also have added other predictable issues with big data, including concerns related to privacy, data protection, and ownership of data [53]. While such conjectural work runs the risk of leading to an ethics of “false alarms,” it also provides an important opportunity to prospectively identify challenges, gather empirical evidence earlier rather than later, and, hopefully, avoid making foreseeable mistakes. To be sure, excessive hype should have no place in either the science of systems medicine or the study of its ethical, legal and societal implications. However, in both of these domains it makes sense to continuously look ahead so we are ready for the future when it inevitably arrives.

## Ethics approval and consent to participate

Not applicable.

## Consent for publication

Not applicable.

## Availability of data and materials

Not applicable.
